# Supplementary material for: Multi‐dataset identification of innovative feature genes and molecular mechanisms in keratoconus
Source: J Cell Mol Med. 2024 Sep 19;28(18):e70079. doi: 10.1111/jcmm.70079 (PMC11412914; doi:10.1111/jcmm.70079)
Supplement: Supplementary file 2 — Material S2. [file JCMM-28-e70079-s002.zip › 7.GSEA analysis/4.MYRF/KEGG_ETHER_LIPID_METABOLISM.html]

Details for gene set KEGG\_ETHER\_LIPID\_METABOLISM[GSEA]

|  || Dataset | MYRF.group.cls#h\_versus\_l |
| Phenotype | group.cls#h\_versus\_l |
| Upregulated in class | l |
| GeneSet | KEGG\_ETHER\_LIPID\_METABOLISM |
| Enrichment Score (ES) | -0.5452095 |
| Normalized Enrichment Score (NES) | -1.3779889 |
| Nominal p-value | 0.09823183 |
| FDR q-value | 0.25908947 |
| FWER p-Value | 0.901 |
Table: GSEA Results Summary

  

Fig 1: Enrichment plot: KEGG\_ETHER\_LIPID\_METABOLISM      
 Profile of the Running ES Score & Positions of GeneSet Members on the Rank Ordered List

  

| SYMBOL | TITLE | RANK IN GENE LIST | RANK METRIC SCORE | RUNNING ES | CORE ENRICHMENT || 1 | PLA2G4E | na | 485 | 0.577 | 0.1093 | No |
| 2 | ENPP6 | na | 4930 | 0.225 | 0.0291 | No |
| 3 | PLA2G3 | na | 8083 | 0.136 | -0.0326 | No |
| 4 | PLA2G12B | na | 10192 | 0.093 | -0.0736 | No |
| 5 | ENPP2 | na | 11117 | 0.076 | -0.0839 | No |
| 6 | PLA2G2D | na | 11306 | 0.073 | -0.0737 | No |
| 7 | PLA2G12A | na | 14161 | 0.036 | -0.1482 | No |
| 8 | AGPS | na | 14243 | 0.035 | -0.1431 | No |
| 9 | PLA2G2C | na | 14507 | 0.032 | -0.1437 | No |
| 10 | PAFAH2 | na | 14554 | 0.032 | -0.1382 | No |
| 11 | PLA2G10 | na | 15631 | 0.022 | -0.1646 | No |
| 12 | PLD1 | na | 17905 | 0.000 | -0.2301 | No |
| 13 | PLA2G6 | na | 17909 | 0.000 | -0.2301 | No |
| 14 | PLD2 | na | 18217 | -0.003 | -0.2384 | No |
| 15 | PLA2G2F | na | 18318 | -0.004 | -0.2405 | No |
| 16 | LPCAT4 | na | 18488 | -0.005 | -0.2443 | No |
| 17 | PLA2G2E | na | 18859 | -0.009 | -0.2530 | No |
| 18 | PAFAH1B1 | na | 19025 | -0.011 | -0.2555 | No |
| 19 | PLA2G7 | na | 20524 | -0.028 | -0.2928 | No |
| 20 | PAFAH1B3 | na | 20618 | -0.029 | -0.2892 | No |
| 21 | JMJD7-PLA2G4B | na | 20701 | -0.030 | -0.2852 | No |
| 22 | PLA2G4A | na | 21803 | -0.043 | -0.3077 | No |
| 23 | PAFAH1B2 | na | 28005 | -0.161 | -0.4521 | No |
| 24 | PLA2G5 | na | 30433 | -0.240 | -0.4709 | Yes |
| 25 | PLA2G2A | na | 33011 | -0.388 | -0.4623 | Yes |
| 26 | LPCAT1 | na | 33682 | -0.467 | -0.3819 | Yes |
| 27 | LPCAT2 | na | 33891 | -0.503 | -0.2806 | Yes |
| 28 | PLA2G1B | na | 34558 | -0.712 | -0.1478 | Yes |
| 29 | CHPT1 | na | 34565 | -0.716 | 0.0047 | Yes |
Table: GSEA details [plain text format]

  

Fig 2: KEGG\_ETHER\_LIPID\_METABOLISM      
 Blue-Pink O' Gram in the Space of the Analyzed GeneSet

  

Fig 3: KEGG\_ETHER\_LIPID\_METABOLISM: Random ES distribution      
 Gene set null distribution of ES for **KEGG\_ETHER\_LIPID\_METABOLISM**
